# Association among gestational diabetes mellitus, periodontitis and prematurity: a cross-sectional study

**DOI:** 10.20945/2359-3997000000435

**Published:** 2022-01-01

**Authors:** Carla Andreotti Damante, Gerson Aparecido Foratori, Paula de Oliveira Cunha, Carlos Antonio Negrato, Silvia Helena Carvalho Sales-Peres, Mariana Schutzer Ragghianti Zangrando, Adriana Campos Passanezi Sant’Ana

**Affiliations:** 1 Universidade de São Paulo Faculdade de Odontologia de Bauru Departamento de Prótese e Periodontia Bauru SP Brasil Departamento de Prótese e Periodontia, Faculdade de Odontologia de Bauru, Universidade de São Paulo, Bauru, SP, Brasil; 2 Universidade de São Paulo Faculdade de Odontologia de Bauru Departamento de Odontopediatria, Ortodontia e Saúde Pública Bauru SP Brasil Departamento de Odontopediatria, Ortodontia e Saúde Pública. Faculdade de Odontologia de Bauru, Universidade de São Paulo, Bauru, SP, Brasil; 3 Universidade de São Paulo Faculdade de Odontologia de Bauru Departamento de Odontopediatria, Ortodontia e Saúde Pública Bauru SP Brasil Faculdade de Medicina, Departamento de Odontopediatria, Ortodontia e Saúde Pública; Faculdade de Odontologia de Bauru, Universidade de São Paulo, Bauru, SP, Brasil

**Keywords:** Gestational diabetes mellitus, premature birth, periodontitis, pregnancy

## Abstract

**Objective:**

Gestational diabetes mellitus (GDM) causes maternal and infant morbidity. Periodontitis is associated with adverse pregnancy outcomes. The aim of this study was to evaluate periodontal status, prematurity and associated factors in pregnant women with and without GDM.

**Subjects and methods:**

This observational cross-sectional study included 80 pregnant women with GDM (G1 = 40) and without GDM (G2 = 40). Demographic and socioeconomic status, systemic and periodontal health condition, prematurity and newborns’ birth weight were analyzed. For bivariate analysis, Mann-Whitney U-test, t test and Chi-squared test were used. Binary logistic regression analyzed independent variables for periodontitis and prematurity (p < 0.05).

**Results:**

Patients from G1 presented lower socioeconomic status, higher weight and body mass index (BMI). Prematurity (G1 = 27.5%; G2 = 2.5%; p < 0.05) and severe periodontitis percentages (G1 = 22.5%; G2 = 0; p = 0.001) were higher in G1 than in G2. Logistic regression analysis showed that household monthly income (OR = 0.65; 95% CI 0.48-0.86; p = 0.003) and maternal BMI (adjusted OR = 1.12; 95% CI 1.01-1.25; p = 0.028) were significant predictors of periodontitis during the third trimester of pregnancy. Presence of GDM remained in the final logistic model related to prematurity (adjusted OR = 14.79; 95% CI 1.80-121.13; p = 0.012).

**Conclusions: Pregnant:**

women with GDM presented higher severity of periodontitis, lower socioeconomic status, higher overweight/obesity and a 10-fold higher risk of prematurity. Socioeconomic-cultural status and BMI were significant predictors for periodontitis, and GDM was a predictor to prematurity.

## INTRODUCTION

During pregnancy, the increase of progesterone, estrogen and other placental-derived hormones leads to several changes in the levels of immunosuppressants and inflammatory mediators (1).

Gestational diabetes mellitus (GDM) is defined as a condition of glucose intolerance that is first diagnosed during the second or third trimester of pregnancy, in which lower glucose levels are necessary than those for the diagnosis of diabetes unrelated to pregnancy (2). It is generally associated with obesity, previous diagnosis of GDM, advanced maternal age and family history of diabetes (3-5).

Periodontitis is a chronic inflammatory disease of the periodontium associated with the local presence of bacteria (6). In this process, bacterial infiltration occurs in the periodontium and the toxins produced locally stimulate a chronic inflammatory response that progressively destroys the periodontal tissues (6). Pregnant women diagnosed with GDM are more likely to present worst periodontal condition (7). Likewise, a systematic review with meta-analysis found a significant association between periodontitis and GDM in four cross-sectional studies and two case-control studies. Nevertheless, the case-control studies showed inconsistent data after sensitivity tests (5). Consequently, current scientific evidence cannot corroborate a positive association between periodontitis and GDM.

GDM is a significant cause of maternal and infant morbidity, including macrosomia and maternal hypertensive disorders (5,8,9). Scientific literature also highlights an association between periodontitis and adverse pregnancy outcomes, such as preterm birth and low birth weight (10,11).

Considering the lack of evidence regarding the association between GDM and periodontitis and the adverse effects of both conditions on neonates’ health at birth, the aim of this study was to evaluate the periodontal status, prematurity and associated factors in pregnant women with and without GDM. The null hypotheses of this study are: (1) there are no changes in the periodontal parameters of women with GDM; (2) the babies of women with GDM are born within the normal period of gestation (after the 37^th^ gestational week). Alternative hypotheses are: (1) there are changes in the periodontal parameters of women with GDM; (2) these women's babies are born prematurely (before the 37^th^ gestational week).

## SUBJECTS AND METHODS

The *Strengthening the Reporting of Observational Studies in Epidemiology* (STROBE) guidelines were used to ensure the accurate reporting of this study (12).

### Ethical aspects

This study received approval from the Ethics Committee on Human Research of Bauru School of Dentistry, University of São Paulo (CAAE 58339416.4.0000.5417). The study was conducted in accordance with the Helsinki Declaration, revised in 2013. All subjects provided written informed consent prior to participating.

### Sample composition

This cross-sectional study was conducted from March 2018 to February 2019. The sample was consecutively recruited by convenience from the public health sector in the city of Bauru, São Paulo, Brazil. This study was composed of 80 patients who were divided into two groups: G1 – pregnant women with GDM (n = 40) and G2 – pregnant women without GDM (n = 40). All patients were evaluated regarding their oral health during the third trimester of pregnancy (27^th^-35^th^ gestational weeks). The presence/absence of GDM was obtained from medical records. The diagnosis of GDM was completed by a 2-hour oral glucose tolerance test performed with 75 grams of glucose, between 24-28^th^ gestation weeks, according to the International Association of Diabetes and Pregnancy Study Group criteria (13). To diagnose GDM, at least one glycemic value ≥92 mg/dL (fasting), ≥180 mg/dL (one hour) and ≥153 mg/dL (two hours) needed to be present (13).

Inclusion criteria for the study were to have had performed the examination of the glycemic curve for the diagnosis of GDM between 24-28^th^ gestational weeks; the absence of systemic diseases; regular gestational follow-up with obstetricians and being in the third trimester of pregnancy. Exclusion criteria were the presence of neuromotor or communication difficulties, use of illicit drugs, consumption of alcohol during pregnancy, smoking, preeclampsia, any severe gestational problem requiring absolute rest, current orthodontic and/or dental treatment or the presence of edentulism.

### Anthropometric measurements

Patients’ weight and height during pregnancy were obtained through an automatic scale (MIC 300PP; Micheletti Ind., São Paulo, São Paulo, Brazil) and stadiometer (2.20; WCS Ind., Curitiba, Paraná, Brazil) located at Bauru School of Dentistry, University of São Paulo. Pre-pregnancy weight and body mass index (BMI) were obtained from medical records. Normal-weight pregnant women were those who had a pre-pregnancy BMI between 18.5 and 24.9 kg/m^2^. Pregnant women were considered overweight when pre-pregnancy BMI was greater than or equal to 25.0 kg/m^2^, and obese when pre-pregnancy BMI was at least 30.0 kg/m^2^ (2,14).

Patients were also classified according to weight gain, as defined by the Institute of Medicine protocol (15). This classification establishes the recommended weight gain during gestation according to patients’ nutritional status found before pregnancy. If the patient gained more than the highest recommended value, she was classified as presenting excessive weight gain.

### General assessments

Socioeconomic status was assessed according to education level and household monthly income, which were graded as follows: 0, illiteracy; 1, did not complete primary education; 2, completed primary education; 3, did not complete high school; 4, completed high school; 5, did not complete higher education; 6, completed higher education; 7, specialization; 8, master's degree; 9, PhD.

Household monthly income was based in the Brazilian minimum wage (MW) (approximately USD 220.00) and categorized in the following levels: level 1 – family receiving up to one MW; level 2 – between 1 and 2 MW; level 3 – between 2 and 3 MW; level 4 – between 3 and 4 MW; level 5 – between 4 and 5 MW; level 6 – family receiving more than 5 MW.

### Periodontal examinations

Oral examinations were conducted by a qualified dentist who was calibrated by a gold standard examiner (kappa intra-examiner = 0.95; 95% confidence interval [CI] = 0.89-0.97; kappa inter-examiner = 0.92; 95% CI = 0.87-0.95). A plain oral mirror n. 05 (Cod. 7503; Duflex/SS White, Juiz de Fora, Minas Gerais, Brazil), a standard Universal North Carolina periodontal clinical probe (QD.320.05; Quinelato, Schobell Ind. Ltda, Rio Claro, São Paulo, Brazil), and a syringe with compressed air were used to examine the oral cavity. All teeth were evaluated, excluding third molars.

Periodontal analysis was performed including probing depth (PD) and clinical attachment loss (CAL). PD was measured from the free gingival margin to the bottom of the periodontal pocket and CAL was measured from the cementoenamel junction to the base of the periodontal pocket (16). Six sites of each tooth were assessed (mesial, center, distal, both in the buccal and lingual surfaces).

Presence of periodontitis was confirmed if interproximal CAL was present at ≥2 non-adjacent teeth, or buccal CAL ≥3 mm with pocketing >3 mm was detectable at ≥2 teeth. Additionally, CAL could not be ascribed to other causes such as: gingival recession of traumatic origin; cervical dental caries, CAL on the distal aspect of a second molar associated with malposition or extraction of a third molar, endodontic lesion and vertical root fracture (17). Staging of periodontitis was classified in I, II and III as previously described (17). Bleeding on probing (BOP) for each assessed site was examined considering the presence or absence of bleeding according to Ainamo and Bay (18).

### Neonates’ data

After labor, patients were contacted in order to obtain neonates’ data. Mothers provided babies’ birth weight and length, as well as type of delivery and date of birth. Data were classified as follows: Low birth weight (LBW) < 2,500 g (19); insufficient weight at birth (IWB) = 2,500 to 2,999 g (19); normal birth weight (NBW) = 3,000 to 3,999 g (20); high birth weight (HBW) or macrosomia > 4,000 g (21). Prematurity was considered present when the birth occurred before 37^th^ gestational weeks.

Based on gender and gestational age at birth, all children who were born prematurely were classified according to their weight for gestational age. For this purpose, the intrauterine growth curve “International Fetal and Newborn Growth Consortium for the 21^st^ Century (INTERGROWTH^21st^)” was used (22). Neonates who presented birth weight below the 10^th^ percentile for their gestational age were considered small for gestational age (SGA); those who presented birth weight above the 90^th^ percentile for their respective gestational age were considered large for gestational age (LGA).

### Statistical analysis

Statistical analysis was performed with IBM SPSS Version 25 (IBM Corp. Released 2017. IBM SPSS Statistics for Windows, Version 25.0. Armonk, NY: IBM Corp.). According to previous evidence in the same field (16,23), the power test was calculated considering an intergroup difference between the mean CAL during pregnancy of at least 10%, with a standard deviation of 10%. Based on the mean CAL and standard deviations of the two groups, an effect size of 0.90 was obtained, resulting in a power of 90.5% with this study's sample size. In addition, for sample size, the Hosmer and Lemeshow protocol for logistic regression analysis was considered (24), which allows the inclusion of 10 cases for each combination of independent variables (25-27). In this study, dichotomization of periodontitis (0 = no periodontitis; 1 = periodontitis) and babies’ prematurity (0 = no prematurity; 1 = prematurity) was performed for binary logistical regressions, and in these models a maximum of four independent variables were inserted. Thus, the inclusion of 80 patients in the sample was considered acceptable.

Thus, statistical analysis was performed in two steps: 1) bivariate analysis and 2) logistic regression by the stepwise backward (likelihood ratio) method. In bivariate analysis, first the Kolmogorov-Smirnov test was applied to verify the normality of the variables, and the following tests were used: Mann-Whitney U-test, t test and Chi-squared test. Binary logistic regression was performed in order to analyze which independent variables could be related to the presence of periodontitis during the third trimester of pregnancy and to prematurity. The independent variables inserted in the initial logistic regression model regarding the presence of periodontitis during pregnancy were: presence of GDM, maternal BMI and household monthly income (Supplementary file 1). The independent variables inserted in the initial logistic regression model regarding the prematurity were: presence of GDM, presence of periodontitis, maternal BMI and household monthly income (Supplementary file 2). Hosmer-Lemeshow, collinearity, and residual analyses were used to increase the understanding of the logistic regression results. A significance level of 5% was adopted.

## RESULTS

**Figure 1:**
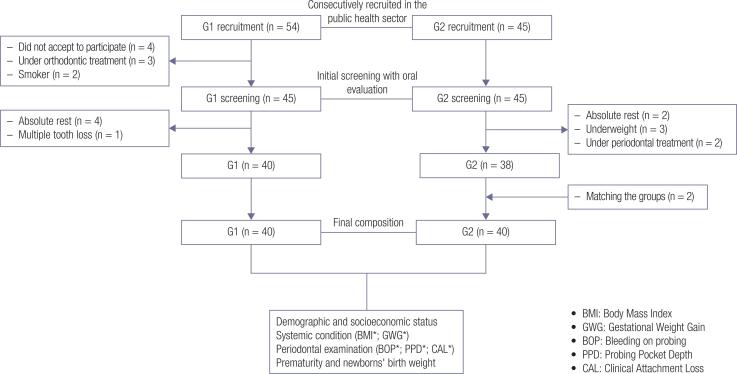
Sample composition.

Initially, 54 pregnant women with GDM were consecutively recruited from specific health care centers for pregnant women with diabetes. Among them, four women did not accept to participate in the study, three of them reported to use orthodontic devices and two were smokers during pregnancy. Therefore, the initial screening with oral evaluation was performed in 45 pregnant women with GDM. Only 41 participants showed up on the day of the consultation to take the research exams. The four missing women justified being on absolute rest during pregnancy. One woman was excluded from the sample for having multiple tooth loss. Simultaneously, 45 pregnant women without GDM were consecutively recruited from public health units in Bauru. All of them underwent the initial screening and were scheduled to take the research exam. However, two of them did not show up, and justified being on rest; three were classified as underweight and two women mentioned being under periodontal treatment recent to that period, so they were excluded from the sample. At that time, in order to match the groups, two pregnant women without GDM were recruited. Finally, the sample consisted of 80 pregnant women divided into: G1 - with GDM (n = 40) and G2 - without GDM (n = 40). Figure 1 shows the flowchart of the sample composition according to STROBE.

Overall, pregnant women from G1 had lower education levels, lower household monthly income (p < 0.0001), greater pre-pregnancy and pregnancy weight, greater pre-pregnancy and pregnancy body mass index (BMI) and greater weight gain during pregnancy in comparison with patients from G2 (Table 1). Prior to pregnancy, 30% (n = 12) of women from G1 were classified as being overweight and 40% (n = 16) as being obese. In contrast, 25% (n = 10) of women from G2 were considered as being overweight, while 12.5% (n = 5) were obese. A higher prevalence of excessive weight gain was found in patients from G1 than in those from G2 (p = 0.0009) (Table 1).

**Table 1:** Comparison of contextual variables between groups

Variables	G1 (n = 40)	G2 (n = 40)	p
Maternal age (years)	32.5 [24.5-36.5]	30 [27.5-33]	0.335*
Education level*	3.5 [1–4]	6 [4–6]	**<0.0001** *
Household monthly income#	2 [1–3]	5.5 [4–6]	**<0.0001** *
Pre-pregnancy weight (kg)	75.64 ± 17.52	65.12 ± 11.14	**0.002** †
Pre-pregnancy BMI (kg/m^2^)	28.77 [24.12-32.95]	24.19 [21.96-27.77]	**0.002** *
Weight during pregnancy (kg)	83.78 ± 15.41	73.10 ± 10.74	**0.0006** †
BMI during pregnancy (kg/m^2^)	32.21 ± 6.13	27.90 ± 3.94	**0.0003** †
Weight gain during pregnancy – n (%)			**0.0009** ‡
Normal	26 (65%)	38 (95%)	
High	14 (35%)	2 (5%)	

Mean ± SD; Median [1^st^-3^rd^ quartiles]; SD: standard deviation; p: significance level; BMI: body mass index; (n): number of patients.

* Variables: Educational level 0 – illiteracy, 1 – incomplete primary education, 2 – completed primary education, 3 – incomplete high school, 4 – completed high school, 5 – incomplete higher education, 6 – completed higher education, 7 – specialization, 8 – master's degree, 9 – PhD.

# Brazilian minimal wage – MW – (USD 220.00) level 1 – up to 1 MW; level 2 – between 1 and 1 MW; level 3 – between 2 and 3 MW; level 4 – between 3 and 4 MW; level 5 – between 4 and 5 MW; level 6 – above 5 MW.

p: * Mann-Whitney U-test;

† *t* test;

‡ Chi-square.

There was no intergroup difference regarding bleeding on probing (p = 0.796), nonetheless, patients from G1 showed higher PD (p < 0.0001) and CAL (p = 0.0001) values. Moreover, 65% (n = 26) of patients from G1 were diagnosed with periodontitis, being 22.5% (n = 9) classified as stage III. In contrast, 32.5% (n = 13) of patients from G2 were diagnosed with stage II periodontitis and 67.5% (n = 27) presented no periodontitis (Table 2). Additionally, of those 28 women in G1 categorized as overweight/obese, 20 had periodontitis (2, 10 and 8 in the stages I, II and III of periodontitis, respectively), whilst of those 15 women from G2 categorized as overweight/obese, nine of them had periodontitis (all in the stage II of periodontitis).

**Table 2:** Comparison of periodontal parameters between groups

Variables		G1 (n = 40)	G2 (n = 40)	p
PD (mm)		2.28 ± 0.49	1.90 ± 0.24	**<0.0001** †
CAL (mm)		2.27 [2.00-2.65]	1.96 [1.86-2.08]	**0.0001** *
BOP (%)		24.99 ± 21.04	25.99 ± 12.46	0.796†
Periodontitis severity – n (%)				
	No	14 (35%)	27 (67.5%)	
	Stage I	2 (5%)	0	**0.001** ‡
	Stage II	15 (37.5%)	13 (32.5%)	
	Stage III	9 (22.5%)	0	

Mean ± SD; Median [1^st^-3^rd^ quartiles]; SD: standard deviation; p: significance level; PD: probing pocket depth; CAL: clinical attachment level; BOP: bleeding on probing; (n): number of patients.

* Mann-Whitney U-test;

† *t* test;

‡ Chi-square.

Regarding neonates’ characteristics, the presence of GDM did not influence birth length, weight and type of delivery. However, women from G1 presented higher prevalence of premature babies (p = 0.001) (Table 3). Of the 11 children who were born prematurely to women from G1, 54.54% (n = 6) were considered LGA according to INTERGROWTH^21st^ (22). The only baby who was born prematurely in G2 had adequate weight for the gestational age at birth.

**Table 3:** Comparison of childbirth data between groups

Variables		G1 (n = 40)	G2 (n = 40)	p
Childbirth height (cm)		48 [46.75-49.75]	49 [47–50]	0.193^*^
Childbirth weight (kg)		3.42 ± 0.65	3.24 ± 0.39	0.137^†^
Delivery type – n (%)				
	Cesarean	33 (82.5%)	30 (75%)	0.415^‡^
Prematurity – n (%)				
	Yes	11 (27.5%)	1 (2.5%)	**0.001** ^‡^

Mean ± SD; Median [1^st^-3^rd^ quartiles]; SD: standard deviation; p: significance level; (n): number of patients.

Binary logistic regression was performed to verify the independent predictors of periodontitis (0 = no periodontitis; 1 = periodontitis) in the studied population (Supplementary file 1). Collinearity analysis showed that all the independent variables inserted in the regression models showed values of tolerance greater than 0.10 and Variance Inflation Factor (VIF) values lower than 10 (VIF < 2). Household monthly income and maternal BMI remained related to periodontitis [X^²^(2) = 22.44; p < 0.0001; Negelkerke's R^²^ = 0.326] in the final logistic regression model. The final model's overall accuracy was 75%. Hosmer and Lemeshow analysis indicated a Chi-square for the final model of 4.32 for 8 degrees of freedom (p = 0.827). Both household monthly income (adjusted OR = 0.65, 95% CI 0.48-0.86, p = 0.003) and maternal BMI (adjusted OR = 1.12, 95% CI 1.01-1.25, p = 0.028) were significantly associated with periodontitis. Household monthly income presented as a negative coefficient, indicating that the lower the household monthly income, the higher was the frequency of periodontitis.

The binary logistic regression was performed to verify the independent predictors of babies’ prematurity (0 = no prematurity; 1 = prematurity) (Supplementary file 2). Collinearity analysis showed that all the independent variables inserted in the regression models showed values of tolerance greater than 0.10 and VIF < 2. Presence of GDM remained in the final logistic model associated with prematurity (adjusted OR = 14.79, 95% CI 1.80–121.13, p = 0.012). The final model [X^²^(1) = 11.22; p = 0.001; Negelkerke's R^²^ = 0.22] showed an overall accuracy of 85%.

## DISCUSSION

This study evaluated the periodontal status and the related factors in pregnant women with and without GDM, and their association with the neonates’ health outcomes. Our main findings suggest that GDM is associated with higher prevalence and severity of periodontitis. Moreover, pregnant women with GDM had a higher percentage of premature babies, despite the babies being born with normal anthropometric parameters.

GDM is associated with advanced maternal age and with the increase in prevalence of obesity found among pregnant women worldwide, representing an important economic burden for the public health care system (28). In this study, there was no difference between groups regarding maternal age, however, 70% of pregnant women from G1 were considered as being overweight or obese (Table 1). This can be explained by the fact that the presence of excessive adiposity is associated with visceral accumulation of adipose tissue, which directly contributes to insulin resistance (29).

Another factor that may be associated with the high prevalence of GDM is excessive gestational weight gain (30). In this study, 35% of patients from G1 (n = 14) presented excessive gestational weight gain, whilst only 5% from G2 presented the same condition (Table 1). We hypothesized that excessive intake of caloric food and abundant nutritional availability during pregnancy, associated with an increase in insulin resistance, can cause an impairment on the glucose metabolism, leading to the onset of GDM (16). These authors also found an association between maternal overweight, excessive gestational weight gain and an increased prevalence of GDM during the second trimester of pregnancy (16).

The association of these risk factors with adverse outcomes is mediated by the contextual variables of each subject. Therefore, socioeconomic and cultural conditions play an important role in the aforementioned outcomes. In this study, patients from G1 showed lower education level and household monthly income (Table 1). Consequently, it is expected that low access to information and to regular health services, as well as poor eating habits and lack of physical activity could result in a higher prevalence of overweight (16) and excessive weight gain during pregnancy (23), which, in turn, are associated with the presence of GDM.

In the present study, patients from G1 had poorer periodontal condition with higher PD and CAL. Our results are in accordance with those from Xiong and cols. demonstrating a higher percentage of periodontitis among patients with GDM (31). Our study showed that 22% of patients from G1 presented severe periodontitis, whilst for G2, 67.5% had no periodontitis and 32.5% had mild periodontitis (Table 2). Women with GDM may be at higher risk of developing more severe periodontal disease than those without GDM, even after delivery (32). Özçaka and cols. observed higher periodontitis rates, plaque accumulation and BOP in patients with GDM compared to those without GDM (33). As aforementioned, there was no intergroup difference for maternal age in this present study. Nonetheless, it is important to point out that periodontal disease is also mediated by patients’ socioeconomic-cultural condition, since low household monthly income and educational level result in inadequate oral hygiene habits and low access to oral health care (34). In this study, the independent variables better associated with the occurrence of maternal periodontitis through the logistic regression model were household monthly income (OR = 0.651; p = 0.003) and high BMI (OR = 1.129; p = 0.028) (Supplementary file 1).

The association between periodontal disease and GDM remains unclear and may be misinterpreted due to a variety of confounding factors. There is a hypothesis suggesting that the levels of hyperglycemia found in GDM may be too mild and too short to have a significant effect on gingival tissues and cause periodontitis (31). Some studies (31-33) suggest that this hypothesis is either not plausible or there might be other factors influencing the worst periodontal status in patients with GDM. Indeed, it is possible that periodontitis can be an etiological factor for GDM instead of a consequence of this condition, since the chronic subclinical inflammation of periodontitis induces local host immune responses and causes transient bacteremia, which may affect the systemic health (31). Maternal gingival inflammation may result in insulin resistance (35), which could exacerbate the physiological insulin resistance during pregnancy, leading to an impairment on glucose tolerance and finally to GDM (33).

A recent study showed that crevicular fluid concentrations of matrix metalloproteinases 8 and 9 (MMP-8 and MMP-9) were increased since the beginning of pregnancy in patients with GDM (36). This increase of inflammatory mediators is also observed in severe periodontitis (36). Moreover, bacterial load may contribute to the worsening of periodontal status, given that there is an association between the severity of periodontitis and higher counts of *Porphyromonas gingivalis* and *Prevotella* *intermedia* in patients with GDM (37).

Obesity also negatively influences the periodontal condition of individuals. Recent studies showed a higher prevalence of periodontitis in overweight pregnant women (16,25-27). It is important to consider that obesity and periodontitis share common modulating factors, such as low socioeconomic and cultural status. In addition, the adipose tissue of overweight and obese individuals secretes inflammatory mediators, which in turn cause an exacerbated inflammatory response in the whole body (38). Therefore, even with a small amount of biofilm on the teeth, there is an exacerbated inflammation in the periodontal tissues of overweight individuals, becoming even more intense due to high levels of estrogen and progesterone found in pregnant women (16). In this study, 20 pregnant women from G1 had both overweight/obesity and periodontitis (n = 2, n = 10 and n = 8 in the stages I, II and III of periodontitis, respectively). In contrast, nine of women from G2 had both overweight/obesity and periodontitis (all of them in the stage II of periodontitis). The high prevalence of pregnant women with overweight/obesity and periodontitis in both groups reflected that maternal BMI remained in the final logistic regression model related to the presence of periodontitis.

There is no consensus regarding the association between GDM, obesity, maternal periodontitis and prematurity. As stated above, there is an association between GDM, obesity and periodontitis. Moreover, medical studies claim that both obesity and GDM are associated with macrosomia due to insulin resistance which results in an increased availability of glucose to the fetus. These elevated levels of glucose cross the placenta, and contribute to fetal hyperinsulinemia and accelerated fetal growth, with babies generally presenting above normal size and weight (39). Also, elevated levels of triglycerides are found in pregnant women with insulin resistance, which are cleaved into smaller molecules and transferred to the fetal circulation, resulting in greater energy input to the fetus (39). In contrast, in literature there is plausible evidence that periodontitis is associated with prematurity and low birth weight (40). The presence of Gram-negative periodontopathogens may impair the development of the fetus directly or indirectly. Periodontal bacteria may lodge in the placenta through the bloodstream, or can indirectly mediate inflammation through cytokines that are released in periodontal tissues. Hence, bacteria prevent adequate absorption of nutrients by the fetuses and stimulate early contractions, with the possibility of premature rupture of membranes (PROM), resulting in prematurity and low birth weight (40).

Jesuino and cols. demonstrated that children who were born from women with excessive gestational weight gain had above normal weight, considering their z-score parameters (23). On the other hand, Foratori-Junior and cols. demonstrated an association between maternal overweight, periodontitis and low birth weight (26). Yet, the association of GDM with prematurity is still unclear. Our results showed that 27.5% of patients from G1 presented preterm birth, whilst only 2.5% from G2 showed the same condition (Table 3). Although there was no intergroup difference regarding the infants’ weight at birth, children who were born premature were categorized more frequently as LGA, according to the INTERGROWTH^21st^ growth curve (22), which takes into account sex, birth weight and gestational week of birth.

Adverse pregnancy outcomes are associated with periodontitis, with an odds-ratio ranging from 1.10 to 20 (41). In addition, there is an association between pre-pregnancy BMI and perinatal outcomes (42). As a variety of factors may be related to preterm birth, the binary logistic regression was performed to verify which independent variable could be a predictor of prematurity. The presence of GDM, and not periodontitis, was related to this outcome, but with a high range of 95% confidence interval, which may be explained by the limitation of this study in respect of the small sample size (OR = 14.79; 95% CI 1.80-121.13; p = 0.012) (Supplementary file 2). We hypothesized that periodontitis did not remain in the final logistic model associated with prematurity in this study due to the small sample size and numerous confounding factors, considering the high prevalence of women with both GDM and overweight/obesity, which might be inversely associated with prematurity.

Our study has some limitations. The cross-sectional design of the study makes it impossible to infer causality (cause and effect) of the study outcomes. The association tests performed in this study using logistic regression models showed low values of odds-ratio, which can be explained by the sample size. As aforementioned, prospective cohorts with a larger number of patients (preferably population-based studies) evaluating the association of the same outcomes are necessary to be more representative in order to extrapolate data to other populations. Moreover, the recruitment of the sample by convenience from public health service in Bauru also is a limitation of this study. Finally, some laboratory analyzes at molecular levels are necessary to better understand the systemic diseases’ effect on periodontium. Consequently, future studies should perform analyses evaluating glycemic control and its relationship with inflammatory mediators in saliva and plasma.

Despite the limitations, our study sheds some light regarding the association between GDM, periodontitis and prematurity. Thus, the authors call the attention of doctors and dentists to the importance of the transdisciplinary and holistic approach of the pregnant woman in order to offer prevention and treatment for these patients and, consequently, improve the health of their children.

In conclusion, pregnant women with GDM presented lower socioeconomic status, higher prevalence of overweight/obesity and higher prevalence and severity of periodontitis. Women's socioeconomic-cultural status and BMI were the factors associated with periodontitis during pregnancy, whilst GDM was the factor associated with pre-term labor.

## Appendix

**Supplementary file 1:** Binary logistic regression model showing independent variables related to periodontitis during the third trimester of pregnancy

		B	df	p	Adjusted OR	95% CI
Model 1	Household monthly income	-0.404	1	0.019	0.66	0.47-0.93
	Maternal BMI	0.119	1	0.034	1.12	1.01-1.25
	Presence of GDM	0.180	1	0.775	1.19	0.34-4.10
	Constant	-1.808	1	0.297	0.16	
Model 2 (Final model)	Household monthly income	-0.430	1	0.003	0.65	0.48-0.86
	Maternal BMI	0.121	1	0.028	1.12	1.01-1.25
	Constant	-1.698	1	0.313	0.183	

B: coefficient; df: degree of freedom; p significance level; OR: odds-ratio; CI: confidence interval.

**Supplementary file 2:** Binary logistic regression model showing independent variables related to babies’ prematurity

		B	df	p	Adjusted OR	95% CI
Model 1	Presence of GDM	2.184	1	0.064	8.88	0.87-89.84
	Presence of periodontitis	0.615	1	0.448	1.84	0.37-9.03
	Household monthly income	-0.157	1	0.528	0.85	0.52-1.39
	Maternal BMI	-0.001	1	0.991	0.99	0.90-1.10
	Constant	-3.172	1	0.150	0.04	
Model 2	Presence of GDM	2.183	1	0.064	8.87	0.88-89.25
	Presence of periodontitis	0.613	1	0.445	1.84	0.38-8.90
	Household monthly income	-0.156	1	0.522	0.85	0.53-1.37
	Constant	-3.189	1	0.049	0.04	
Model 3	Presence of GDM	2.472	1	0.023	11.84	1.39-100.36
	Presence of periodontitis	0.797	1	0.291	2.21	0.50-9.72
	Constant	-3.993	1	<0.001	0.01	
Model 4 (Final model)	Presence of GDM	2.694	1	0.012	14.79	1.80-121.13
	Constant	-3.664	1	<0.001	0.02	

B: coefficient; df: degree of freedom; p significance level; OR: odds-ratio; CI: confidence interval.
